# Protocol for qRT-PCR analysis from formalin fixed paraffin embedded tissue sections from diffuse large b-cell lymphoma: Validation of the six-gene predictor score

**DOI:** 10.18632/oncotarget.13066

**Published:** 2016-11-04

**Authors:** Nilgun Tekin, Nader Omidvar, Tim Peter Morris, Paulette Conget, Flavia Bruna, Botond Timar, Eva Gagyi, Ranjan Basak, Omkar Naik, Chirayu Auewarakul, Narongrit Sritana, Debora Levy, Juliano Julio Cerci, Sergio Paulo Bydlowski, Juliana Pereira, Mark Pierre Dimamay, Filipinas Natividad, June-Key Chung, Nevin Belder, Isinsu Kuzu, Diana Paez, Maurizio Dondi, Robert Carr, Hilal Ozdag, Rose Ann Padua

**Affiliations:** ^1^ Ankara University, Biotechnology Institute, Ankara, Turkey; ^2^ Department of Hematology, University of Cardiff School of Medicine, UK; ^3^ Medical Research Council (MRC) Clinical Trials Unit at University College (UCL), London, UK; ^4^ Facultad de Medicina Clínica Alemana - Universidad del Desarrollo Santiago, Chile; ^5^ Department of Pathology and Experimental Cancer Research, Semmelweis University, Budapest, Hungary; ^6^ Departments of Medical Oncology & Pathology, Tata Memorial Hospital, Mumbai, India; ^7^ Chulabhorn Cancer Centre and Faculty of Medicine Siriraj Hospital, Bangkok, Thailand; ^8^ Laboratory of Genetics and Molecular Hematology (LIM31), University of São Paulo School of Medicine, São Paulo/SP, Brazil; ^9^ Department of Nuclear Medicine, Quanta - Diagnóstico e Terapia, Curitiba, Brazil; ^10^ Research and Biotechnology Division, St Luke's Medical Centre, Manila, Philippines; ^11^ Department of Nuclear Medicine, Seoul National University Hospital, Seoul, South Korea; ^12^ Department of Pathology, Ankara University School of Medicine, Ankara, Turkey; ^13^ Department of Nuclear Sciences and Application, Division of Human Health, International Atomic Energy Agency, Vienna, Austria; ^14^ Department of Haematology, Guy's & St Thomas' Hospital, King's College, London, UK; ^15^ Institut National de la Sante et de la Recherche Médicale (INSERM) Unité 1131, Université Paris-Diderot, Institut Universitaire d'Hématologie, Hôpital Saint-Louis, Paris, France

**Keywords:** diffuse large B-cell lymphoma (DLBCL), 6-gene predictor score, formalin fixed paraffin embedded tissue (FFPE)

## Abstract

As a part of an international study on the molecular analysis of Diffuse Large B-cell Lymphoma (DLBCL), a robust protocol for gene expression analysis from RNA extraction to qRT-PCR using Formalin Fixed Paraffin Embedded tissues was developed. Here a study was conducted to define a strategy to validate the previously reported 6-gene (*LMO2, BCL6, FN1, CCND2, SCYA3* and *BCL2*) model as predictor of prognosis in DLBCL. To avoid variation, all samples were tested in a single centre and single platform. This study comprised 8 countries (Brazil, Chile, Hungary, India, Philippines, S. Korea, Thailand and Turkey). Using the Kaplan-Meier and log rank test on patients (n=162) and two mortality risk groups (with those above and below the mean representing high and low risk groups) confirmed that the 6-gene predictor score correlates significantly with overall survival (OS, p<0.01) but not with event free survival (EFS, p=0.18). Adding the International Prognostic Index (IPI) shows that the 6-gene predictor score correlates significantly with high IPI scores for OS (p<0.05), whereas those with low IPI scores show a trend not reaching significance (p=0.08). This study defined an effective and economical qRT-PCR strategy and validated the 6-gene score as a predictor of OS in an international setting.

## INTRODUCTION

Curing cancer has become a global priority. Optimizing cure rates requires accurate diagnosis for the selection of the most appropriate treatment, and increasingly this involves genetic subtyping of the disease for both diagnosis and prognostic stratification. Global gene expression alteration is common and found to be correlated with the clinical outcome of cancer. Identification of genes with altered expression level provides valuable information regarding the diagnosis, prognosis and treatment of cancer. Recently high throughput, genome-wide expression profiling (GEP) analyses indicated that some of these genes can be used as marker genes providing important diagnostic and prognostic information about the disease [1, 2].

Diffuse large B-cell lymphoma (DLBCL) is the most frequent lymphoma in adults worldwide. Patients with DLBCL can be risk-stratified on the basis of their clinical characteristics, biological sub classification of the tumor based on gene or antigen expression, and speed of response to treatment based on positron emission tomography (PET) monitoring. Lossos et al. using a univariate analysis of microarray GEP data, identified 6 genes (*LMO2, BCL6, FN1, CCND2, SCYA, BCL2*) from a potentially informative 36 gene panel whose expression had been reported to predict survival [3]. By analyzing the expression of these 6 genes by RT-PCR in 66 patients at the time of diagnosis, they showed that the activation status of these genes correlated with patient overall survival (OS), and was independent of the International Prognostic Index (IPI) score. When this 6-gene data was combined with the clinical IPI at diagnosis, a high predictive index of outcome was achieved. This resulted in a 6-gene model designed to predict outcome in individual patients independent of the IPI, which was validated in two datasets [4, 5]. However, the risk stratification data reported had been generated from Western populations. It is unclear how applicable this would be to predicting clinical outcomes in non-European, non-US populations [6].

As the primary purpose of this project was to inform management of DLBCL in middle income countries across diverse ethnic populations, the first step of this study was to identify an optimum and robust methodology from RNA extraction to real-time quantitative reverse-transcription (qRT-PCR) analysis, for routine cancer gene expression profiling. A gene expression analysis protocol starts with RNA isolation and extends to the analysis of qRT-PCR data. qRT-PCR is the most sensitive method for quantifying gene expression. The robustness of this technique is directly correlated with the integrity and quality of RNA, the choice of detection chemistry (hydrolysis probes, fluorescent hairpins or intercalating dyes) and methods to analyse the data (relative vs. absolute). Thus, a robust gene expression analysis protocol from RNA isolation to qRT-PCR is crucial.

To this end, we investigated different methodologies for quantifying informative gene expression in DLBCL. We used the validated 6-gene model to measure whether the methods compared achieved equivalent outputs and assessing whether the 6-gene model was applicable in an international setting.

## RESULTS

### RNA isolation

The purity of the extracted RNA from formalin fixed paraffin embedded (FFPE) tissues was assessed by OD 260/280 with minimum and maximum values 1.52 and 2.16 respectively. Minimum and maximum RNA concentration were determined as 47.29 ng/μl and 4385.38 ng/μl respectively.

### High volume vs low volume cDNA synthesis comparisons

The workflow for protocol optimization is illustrated (Figure S1). In this study in order to evaluate the impact of cDNA synthesis efficiency on the relative quantification results two cDNA synthesis protocols were followed. The first protocol was the one recommended in the ABI High Capacity cDNA Reverse Transcription Kit. The synthesis volume of this protocol was 20 μl, which was then diluted 10 times with sterile ddH_2_O. The modified protocol synthesis volume is 100 μl. Both cDNA synthesis protocols starting RNA amount was 1000 ng. The number of samples processed with both protocols and both chemistries was shown (Supplementary Tables S1 and S2).

**Figure 1:**
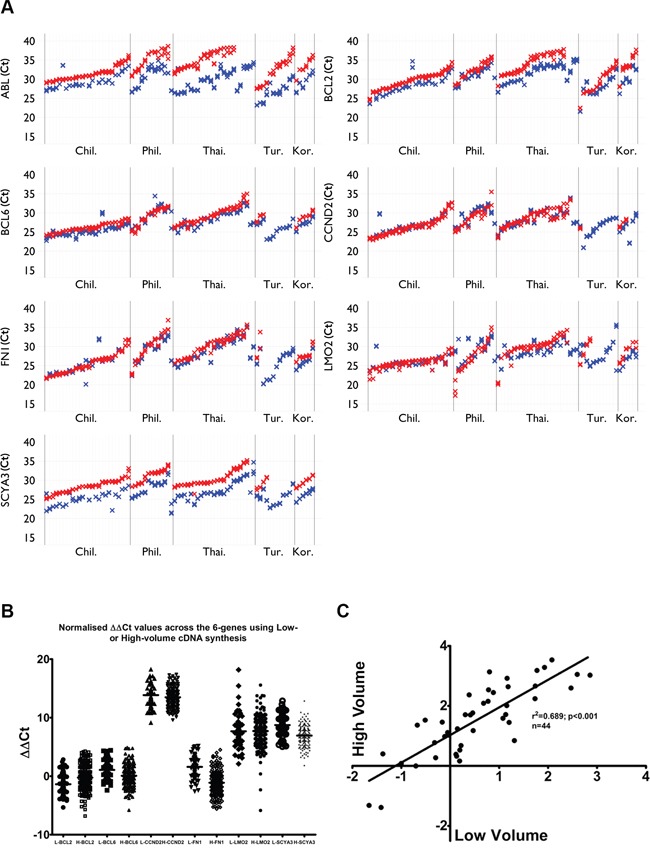
Low versus high volume cDNA synthesis **A.** High volume (blue) consistently gives lower Ct values than low volume (red) for most genes and most samples using Taqman chemistry; **B.** Normalized delta delta Ct values show that low and high volume datasets are equivalent (n=44); **C.** Positive correlation was observed in samples where both low- and high volumecDNA synthesis was applied (p<0.001, n=44).

In order to assess the efficiency, reproducibility and robustness of these two cDNA synthesis protocols the triplicate Ct measurement was analyzed using linear mixed models, and using the graphics described in the methods section. The high volume cDNA synthesis method consistently gave lower Ct with lower variation within the triplicates for *ABL, BCL2, BCL6, CCND2, FN1, LMO2* and *SCYA3* using the Taqman chemistry (Figure 1A). The delta delta Ct values for the 6 genes and 6-gene scores of low and high volume synthesis show that they are equivalent after normalizations to the housekeeping gene ABL and Raji/NLN (Figure 1B and 1C).

### Taqman vs SYBR green comparison

Amplifications of the same targets using Taqman or SYBR Green chemistries were compared (Figure S2). The SYBR Green and Taqman chemistries with the calculated data according to the expression ratio based on efficiency corrected Delta Ct [7] using the plasmid standard curve efficiency show a good correlation (Figure S2A and S2B); the Ct values are concordant between the two chemistries (Figure S2C).

### Standard curves

Expression ratio based on efficiency corrected Delta Ct [7] were separately calculated with the efficiency values obtained from the Raji cell line, plasmid and Normal Lymph Nodes (NLN) standard curves for the each gene. Comparisons of plasmid vs the Raji cell line or plasmid vs NLN standard curves were undertaken by plotting one measurement against the other, and then using plotting the limits of agreement. There was a high agreement between the three different templates (Figure S3 and S4). While using the standard curve from the serial dilutions of the Raji cell line, the standard curve for *FN1* and *CCND2* genes could not be generated due to high Ct values (above Ct35) because of their low expression in the Raji cell line. There was a high compatibility between the Raji cell line and plasmid standard curves for the genes *BCL2, LMO2, BCL6* and *SCYA3* (Figures S4A and S4B). In contrast to standard curves from the Raji cell line, standard curves for the 6 genes were successfully generated from the NLN cDNA. There was a high correlation between NLN and plasmid standard curves for the six genes (Figures S4C and S4D).

### Copy number vs Ct based calculation comparison

Relative expression was calculated using the same data set and three different methods namely: copy number ratio, delta delta Ct, Pfaffl's equation with using the efficiency of plasmid DNA [7]. Comparisons show that all three methods are concordant (Figure S5). There was a high compatibility between Delta Delta Ct and Copy number ratio (Figure S5A and S5B). The compatibility between Pfaffl's equation and Copy number ratio is shown (Figure S5C and S5D).

### Validation of 6-gene model

**Figure 2:**
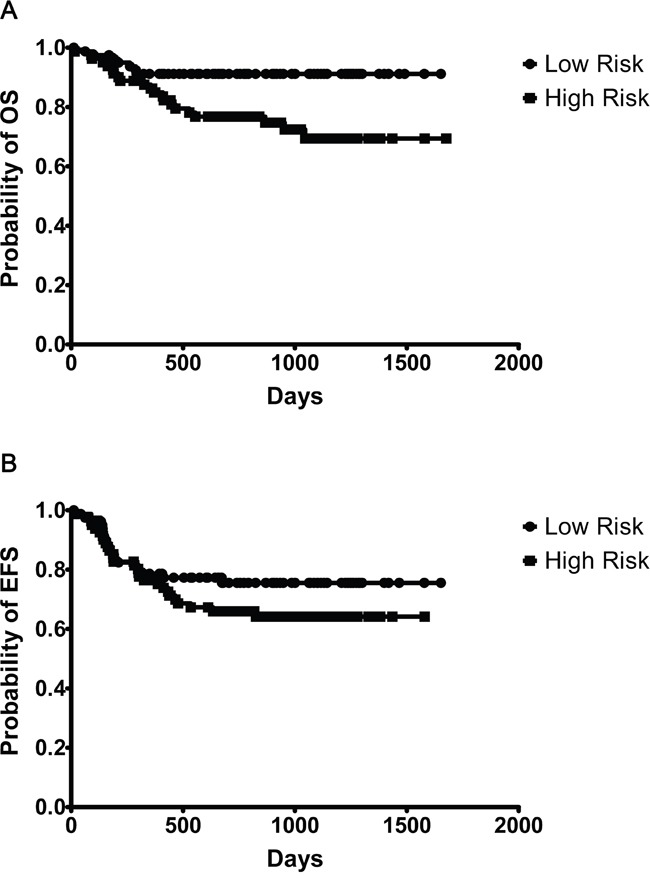
Six-gene model predicts overall survival (OS) in diffuse large B-cell lymphoma (DLBCL) patients treated with Rituximab, cyclophosphamide, doxorubicin (hydroxydaunomycin), vincristine (Oncovin ®), prednisolone (a steroid) (R-CHOP) and CHOP chemotherapy within an international multi-centre setting Kaplan-Meier curves of 162 patients. **A.** show significant extended OS of low risk patients as defined by 6-gene predictor score (p<0.01; patient numbers Low n=81, High n=81); **B.** show no significant EFS of low risk patients as defined by the 6-gene predictor score (p=0.18; patient numbers Low n=81, High n=81).

**Figure 3:**
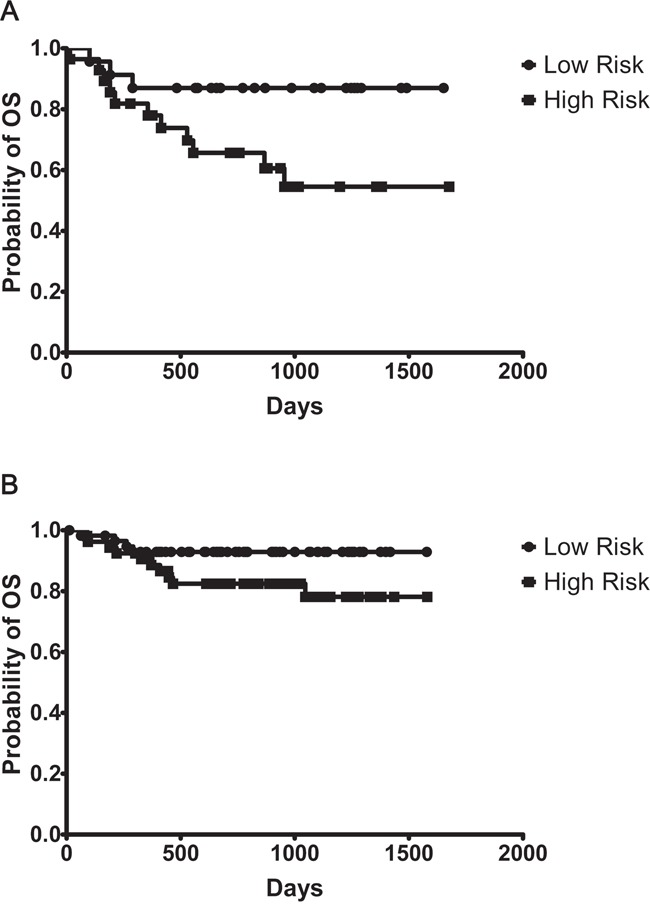
Six-gene predictor model is independent of the International Prognostic Indicator (IPI) **A.** Significant difference is observed in Kaplan-Meier curves of overall survival (OS) based on 6-gene predictor score in high clinical risk patients (IPI score 3-5, n=51) (p<0.05); Patient numbers Low n=23, High n=28; **B.** no significant difference in Kaplan-Meier curves of OS in low clinical risk patients (IPI score 0-2, n=111) (p=0.08); Patient numbers Low n=58, High n=53.

The ‘mortality predictor’ score as described by Lossos et al and modified by Malumbres et al for FFPE sections, based on *LMO2, BCL6, FN1, CCND2, SCYA3*, and *BCL2*, was applied to patients in our study [3]. A positive correlation was observed in the predictor score where both low and high volume data was available (Figure 1C; r^2^=0.689, p<0.001, n=44). The expanded data set incorporating multiple imputational analysis was next employed, where the overall-survival returned a hazard ratio of 0.35 (95% CI 0.17–0.74). This demonstrates that the score predicts relative survival well. The patients were ranked according to mortality predictor score and were divided in high (n=81), and low risk (n=81) groups based on the weighted expression of the 6 genes [8]. The 6-gene predictor model showed that OS was significantly longer in the low risk group as compared to that of the high risk (Figure 2A, p<0.01); whereas EFS was not statistically significantly different between the two risk groups (Figure 2B; p=0.18). To evaluate the prognostic value of the 6-gene model we next performed survival analysis in patients with high and low IPI scores. The OS of high risk vs low patients as defined by the 6-gene score, was significantly different in patients defined as high risk by the IPI (Figure 3A; p<0.05), but not in low risk IPI patients (Figure 3B; p=0.08). No difference was observed within our cohort between CHOP versus R-CHOP treatment regimens in terms of OS (Figure S6A; p=0.41) or EFS (Figure S6B; p=0.66). Furthermore, within the R-CHOP treated cohort, we did not observe a significant difference between the low- versus high- risk groups in terms of OS (Figure S7A; p=0.06) or EFS (Figure S7B; p=0.19). Hans algorithm where available are shown in Table 1; no significant difference was found between GCB vs non-GCB in our cohort (OS p=0.65; EFS p=0.92).

**Table 1:** Patient and treatment details

	TOTAL
Number of Patients	**162**
Sex M	**83** **(51%)**
Age at Diagnosis *Median* (*quartiles*)	**55** **(46,66)**
IPI Score 0-1 2 3 4-5	**75 (46%)** **37 (23%)** **33 (20%)** **17 (11%)**
GCB/non-GCB/unknown (% non-GCB where known)	**56/76/30 (47%)**
Mean 2y Outcomes (days)[range] (95% CI)	**OS: 538** **[480–596]** **EFS: 423** **[366–481]**
R-CHOP CHOP	**134 (83 %)** **28 (17 %)**
Chemotherapy Cycles: <6 6 >6	**19 (12 %)** **84 (52 %)** **59 (36 %)**
Consolidation Radiotherapy Total (Bulky disease) (Non-bulky site)	**42 (28 %)** **(38)** **(4)**
Number of patients with significant Rx delays or dose reductions	**23/100 (23 %)** **(48 not known)**

rituximab, cyclophosphamide, doxorubicin (Hydroxydaunomycin), vincristine (oncovin ®), prednisolone (a steroid) (R-CHOP).

## DISCUSSION

The aim of this international study was to define a robust, sensitive and reproducible strategy to identify the expression profile of the 6-gene model and to validate the six-gene predictor score for outcome in DLBCL. Due to the increasing use of qRT-PCR in the diagnostic and prognostic setting and the necessity to use FFPE tissue for analysis we have developed a robust protocol. It is important to optimize the cDNA synthesis step and to note that most manufacturers recommend low volume cDNA synthesis method. It would appear that, due to the reproducibility of the triplicates and reduced Ct values of high volume cDNA synthesis, the high volume synthesis is superior to the low volume synthesis using the Taqman chemistry (Figure 1). This profile is lost with the SYBR Green reactions possibly due to the larger amplicon size. This is particularly important when dealing with the low quality of RNA we expect from paraffin-derived tissues. However, with the NLN derived RNA, which would be of good quality, the high volume cDNA consistently gave lower Ct values for all of the amplicons tested. However, normalizations ensured that the delta delta Ct values and 6-gene scores were finally equivalent.

The two most commonly used chemistries of Taqman and SYBR Green correlate well. The difference in cost between these reagents has precluded the use of Taqman reagents in resource poor settings; this study shows that SYBR Green method can substitute for Taqman well. However, the optimal amplicon size for SYBR Green is around 300 bp whereas for Taqman it is 100 bp, therefore for FFPE derived degraded RNA Taqman is the chemistry of choice both for size and specificity with the use of an internal probe.

Standard curves generated from the plasmid and cellular derived cDNAs are equivalent and therefore the copy number method, which relies on generating a plasmid derived standard curve correlates with the delta delta Ct and efficiency corrected Ct method. This study shows that it is not necessary to clone every target under investigation for obtaining relative quantities and comparing data from different plates; one can use standard curves derived from a reference cell line. However, to obtain absolute values, it remains necessary to use plasmid derived standard curves. At the end of these optimization steps we concluded that high volume cDNA synthesis, use of Taqman chemistry should be the protocol to follow in especially FFPE derived RNA samples. Here in this international study we have developed a robust, sensitive and reproducible qRT-PCR strategy suitable for the limited budgets available in middle-income countries, from RNA extraction up to data analysis to identify the expression profile of the six gene model.

As with the Malumbres study, where FFPE tissue was used, this international study returned a significant association between the 6-gene predictor score and OS. When added to IPI the 6-gene score was significantly associated with high IPI score and not low IPI score, whereas the Malumbres study was significantly associated with low IPI and not high [8]. This suggests that both studies would benefit from increased sample size. In an international setting involving patients from 8 countries, 3 continents and different ethnic backgrounds our study has demonstrated that the 6 gene score can add some prognostic information to patients with a high clinical risk index, validating this model as a prognosticator in DLBCL, illustrating the utility of molecular expression studies.

## MATERIALS AND METHODS

### Patients

The International Atomic Energy Agency (IAEA) sponsored a prospective study of DLBCL in countries from 8 United Nations geographical regions. Patient samples were obtained from clinical centres in the countries detailed in Table 1. The local ethics committee approved the study and informed consent was obtained from all patients. Consented patients with DLBCL (n=162) in Brazil (n=18), Chile (n=27), Hungary (n=28), India (n=27), South Korea (n=7), Philippines (n=13), Thailand (n=27), and Turkey were treated with a mixture of rituximab, cyclophosphamide, doxorubicin (hydroxydaunomycin), vincristine (Oncovin ®), prednisolone (a steroid) (R-CHOP) (83%) or CHOP (17%) between 2008 and 2013. Diagnosis was made by country representative pathologists and the diagnostic criteria was set up in a collaborative meeting (Ankara). Hans algorithm was used for immunohistochemical subclassification (CD10, Bcl6, MUM1). This method and markers are accepted as gold standard and reliable for the subclassification. These patients were a subset of the cohort of a Coordinated Research Project funded by the IAEA where the use of interim PET was investigated in order to risk stratify patients [9]. CD20 immunohistochemistry was performed for all of the cases for indication of anti CD20 therapy.

### RNA isolation

Total RNA was extracted from FFPE tissues (five sections (10 μm thickness) using Qiagen FFPE Rneasy Kit (Qiagen Cat #74404, Valencia, CA, USA) with a modified deparaffinization step [10]. Briefly, sections were deparaffinized by 2 repeated incubations in xylene for 10 minutes, followed by 2 repeated incubations in 100% ethanol for 5 minutes and then were washed with distilled water for 30 seconds. After deparaffinization, the remaining steps of RNA extraction were followed according to the Qiagen FFPE RNeasy Kit manual. All RNA samples were analyzed spectrophotometrically on a Nanodrop (NanoDrop 1000 Spectrophotometer V3.7, Thermo Fisher Scientific Inc, Wilmington, DE, USA).

### cDNA synthesis

Two different cDNA synthesis methods were evaluated. The integrity of the cDNA templates was evaluated using the Fusion Quant^®^ standard ABL (reference: CGRS-01-4, Ipsogen, France) and subsequently qRT-PCR was carried out on the 7 target genes with Taqman and in a subset, SYBR Green chemistry (Table S1 and Table S2).

#### Molecular cloning of the target genes

The target genes (*LMO2, BCL6, FN1, CCND2, SCYA3*) were amplified (primer details in Supplementary Table S3) and cloned into the TOPO^®^ vector and transformed to chemically competent *E.coli* cells (provided by the Kit) according to the manufacturer's instructions (Invitrogen TOPO TA Cloning Kit Cat No.K4510-20, Carlsbad, CA, USA). Plasmid DNA was purified with QIAprep Spin Miniprep Kit (Cat No.27104 Hilden, Germany) according to the manufacturer's instructions. To confirm the cloning, PCR reactions were repeated for target genes from plasmid DNA minipreps and then sent for sequencing. The *BCL-2* plasmid was already available [11].

#### Low volume

1000 ng RNA was used to synthesize using the ABI High Capacity cDNA Reverse Transcription Kit (Cat. 4368813 Foster City, CA, USA) in a total volume of 20 μl. This cDNA sample was then diluted to 100 μl and 2 μl (20 ng) is used per each qRT-PCR reaction (according to the kit user instructions). Samples from Brazil were synthesized only with the low volume protocol.

#### High volume

1000 ng RNA was used to synthesize using ABI High-Capacity cDNA Reverse Transcription in a total volume of 100 μl. The reaction volume was equal to 5 times of the original reaction mix with 1000 ng RNA. 2μl (20 ng/μl) cDNA samples directly used per each qRT-PCR reaction.

### qRT-PCR analysis

Standard curves were prepared from plasmids containing cloned target genes (*BCL-2, LMO2, BCL6, FN1, CCND2, SCYA3*) that were amplified (method details in supplementary methods information, cloning primer details in Table S3 and SYBR green primers in Table S4). Taqman qRT-PCR assays were conducted on a Light Cycler 480 (Roche, Mannheim, Germany) platform using Life Technologies Applied Biosystems Taqman Gene Expression Assays according to the kit user instructions (BCR ABL1 Hs99999002_mH, BCL2 Hs00153350_m1, BCL6 Hs00277037_m, CCND2 Hs00277041_m1, FN1 Hs00365058_m1, LMO2 Hs00277106_m, SCYA3 Hs00234142_m1, TNFRSF9 Hs00155512_m1) and TaqMan® Universal PCR Master Mix (Applied Biosystems, Foster City, CA, USA) Cat. 4304437). Expression ratios were calculated based on delta delta Ct: R=2^(−ΔΔCT)^ [7].

#### Copy number of the plasmids

Dilutions were calculated for copy numbers of 10^8^ – 10^3^ for each of the 8 plasmids [12, 13]. All qRT-PCRs were undertaken using a 384-plate Roche LC480 instrument (Roche, Mannheim, Germany) at the Ankara University Biotechnology Institute Central Laboratory by one investigator. Most of RNA extractions and the cDNAs were done at Ankara University by the investigators from the participating countries. qRT-PCRs were done from plasmid DNAs of the target genes to generate standard curves with the Power SYBR® Green PCR Master Mix (Life Technologies, Applied Biosystems, Cat.No. 4367659, Carlsbad, CA) according to the manufacturer's instructions.

Formula;

$$N=\frac{Cplasmid}{A}\cdot 1,07085.10^{15}$$

**N:** Copy number (copies/μl)

**C plasmid:** Concentration of the plasmid DNA (μg/μl)

**A:** Size of the plasmid with insert (bp)

#### Standard curves

Standard curves were derived from plasmids, Raji cell line and a normal lymph node (NLN) pool from FFPE sections. For plasmids serial dilutions from 10^8^ to 10^3^ copies were prepared. For the Raji and NLN 6x 10 fold serial dilutions were prepared from the original cDNA stock (1 μg RNA in 100 μl synthesis reaction for high volume and 1 μg RNA in 20 μl for low volume). qRT-PCR reactions were performed.

#### SYBR green reactions

Target genes *BCL2, BCL6, CCND2, FN1, LMO2, SCYA3* and *ABL* qRT-PCR analysis were conducted on a Light Cycler 480 platform using ABI Power SYBR Green PCR Master Mix (Applied Biosystems Cat. 4367659, Foster City, CA, USA). Primer sequences, annealing temperatures and amplicon sizes for the seven target genes and ABL housekeeping gene [14, 15] are listed in Supplementary Table S3. Briefly PCR conditions are 94^°^C for 3 min, 30 cycles of 94^°^C 30 sec, appropriate annealing temperature for 30 sec, and 72^°^C for 30 sec. Followed by a melting program.

### Expression ratios and statistical analysis

Relative quantifications were carried out using three different approaches detailed below and comparative analyses were undertaken.

***Expression Ratio Based on Normalized Copy Numbers*** [16]

$$\begin{array}{lll} R & = & \frac{\text{Patient Target Gene Copy No}}{\text{Patient ABL Copy No}}/\\ & & \frac{\text{Control Target Gene Copy No}}{\text{Control ABL Copy No}} \end{array}$$

***Expression Ratio Based on Efficiency Corrected Delta Ct*** [7]

$$R=\frac{E_{\text{Target Gene}}{}^{\left(ControlCt-PatientCt\right)}}{E_{ABL}{}^{\left(ControlCt-PatientCt\right)}}$$

E: Efficiency

Ct: threshold cycle

***Expression Ratio Based on Efficiency Delta Delta Ct*** [17]

*R* = 2^(−ΔΔ*CT*)^

### Validation of the 6-gene model

As an independent study with measurements on the six-genes score used by Lossos et al and modified by Malumbres et al (*LMO2, BCL6, FN1, CCND2, SCYA3* and *BCL2*) validation of the scores was performed [3]. For the prognostic score analysis samples with ABL Ct values >37 were not included. This cut off was determined from standard curves generated with target genes cloned into plasmids by running an absolute quantification. Thus, as seen from the Figure S8 the log copy number of ABL of the samples at this low range correlates with ABL Ct. Ct 37 corresponds to 9-15 copies, which is within the limits of detection for this assay, which has a cut off of <3 copies [18–20]. As raw Ct values may vary between samples, delta Ct values were compared to FFPE normal lymph node samples. The samples acceptance criteria was based upon the Minimum Information for Publication of Quantitative Real-Time PCR Experiment) (MIQE) guidelines [18] as well as the determined PCR efficiency for each assay using FFPE NLN for ABL and all six genes (all 95.5%-99.5%).

As the original scores were based on data using low volume cDNA synthesis, the initial analysis using separate multiple imputation of low-volume & high-volume data and the Lossos predictor formula was undertaken (n=162 patients) [3, 21]. The normalized gene-expression values were log-transformed (on a base of 2) and the mortality predictor score was calculated based on the following equation: Mortality Predictor Score = (−0.0273xLMO2) + (−0.2103xBCL-6) + (−0.1878xFN1) + (0.0346xCCND2) + (0.1888xSCYA3) + (0.5527xBCL-2) [3]. This score was categorically ranked which allowed their division into two mortality risk groups with those above and below the mean representing high and low risk groups [8]. Unlike the original Lossos study, where fresh tissue was used, the Malumbres method used FFPE tissue, similar to this current study. OS was defined as the time interval between the date of diagnoses to the date of death or last follow-up. Event free survival was defined as the time interval between the date of initial diagnosis and date of disease progression or death from any cause, whichever came first, or date of last follow-up evaluation. Survival curves were estimated using the product-limit method of Kaplan-Meier and were compared using the log-rank test. Multivariate regression analysis according to the Cox proportional hazards regression model [22] with OS or EFS as the dependent variables, was used to adjust for the effect of the Mortality Predictor Score and IPI. The t-test or Pearson's Chi-squared test were used to compare the clinical characteristics between the low and high risk patient groups of the 6-gene model. p < 0.05 was considered significant. Statistical analyses were carried out in Stata, GraphPad Prism and SPSS.

## SUPPLEMENTARY FIGURES AND TABLES


