# Supplementary material for: Protocol for qRT-PCR analysis from formalin fixed paraffin embedded tissue sections from diffuse large b-cell lymphoma: Validation of the six-gene predictor score
Source: Oncotarget. 2016 Nov 4;7(50):83319–29. doi: 10.18632/oncotarget.13066 (PMC5347772; doi:10.18632/oncotarget.13066)
Supplement: Supplementary file 1 [file oncotarget-07-83319-s001.pdf]

Protocol for qRT-PCR analysis from formalin fixed paraffin embedded tissue sections from diffuse large b-cell lymphoma: Validation of the six-gene predictor score

SUPPLEMENTARY FIGURES AND TABLES

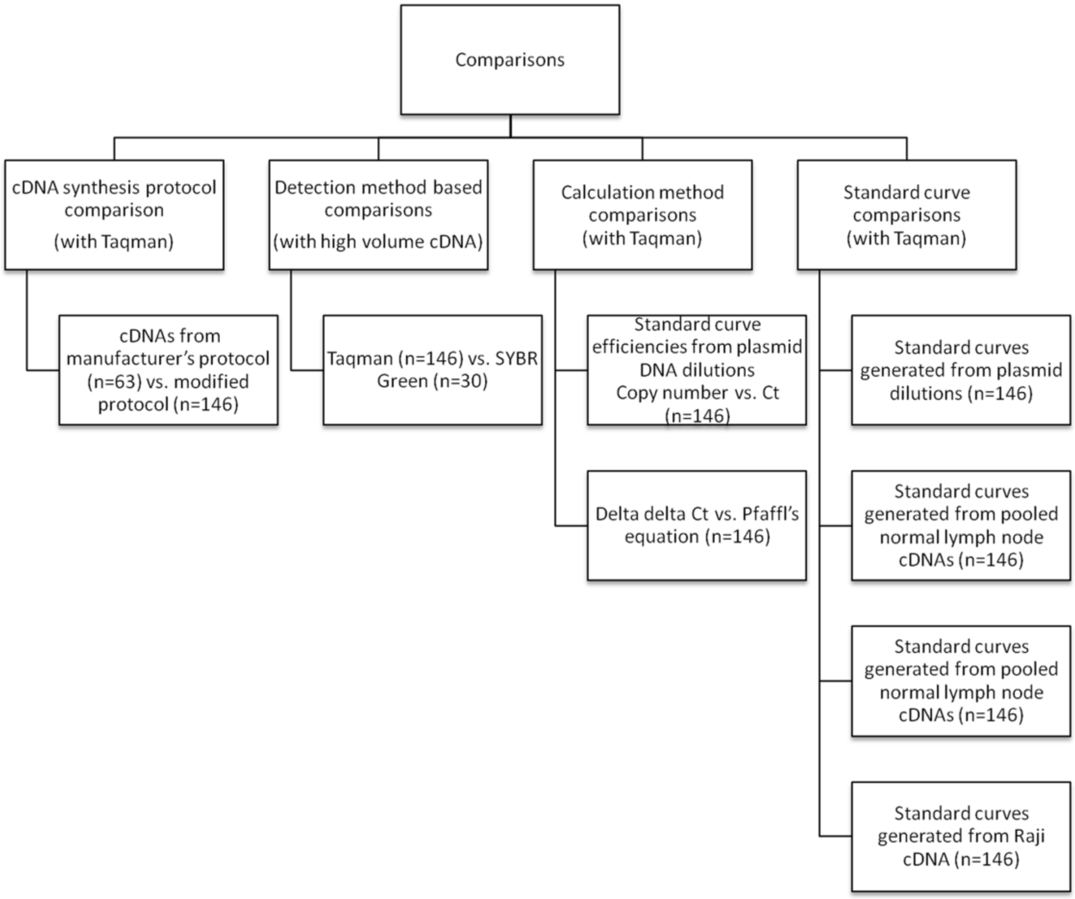

Supplementary Figure S1: Comparison workflow. Samples with Cts above 35 for ABL were not included for methods analyses.

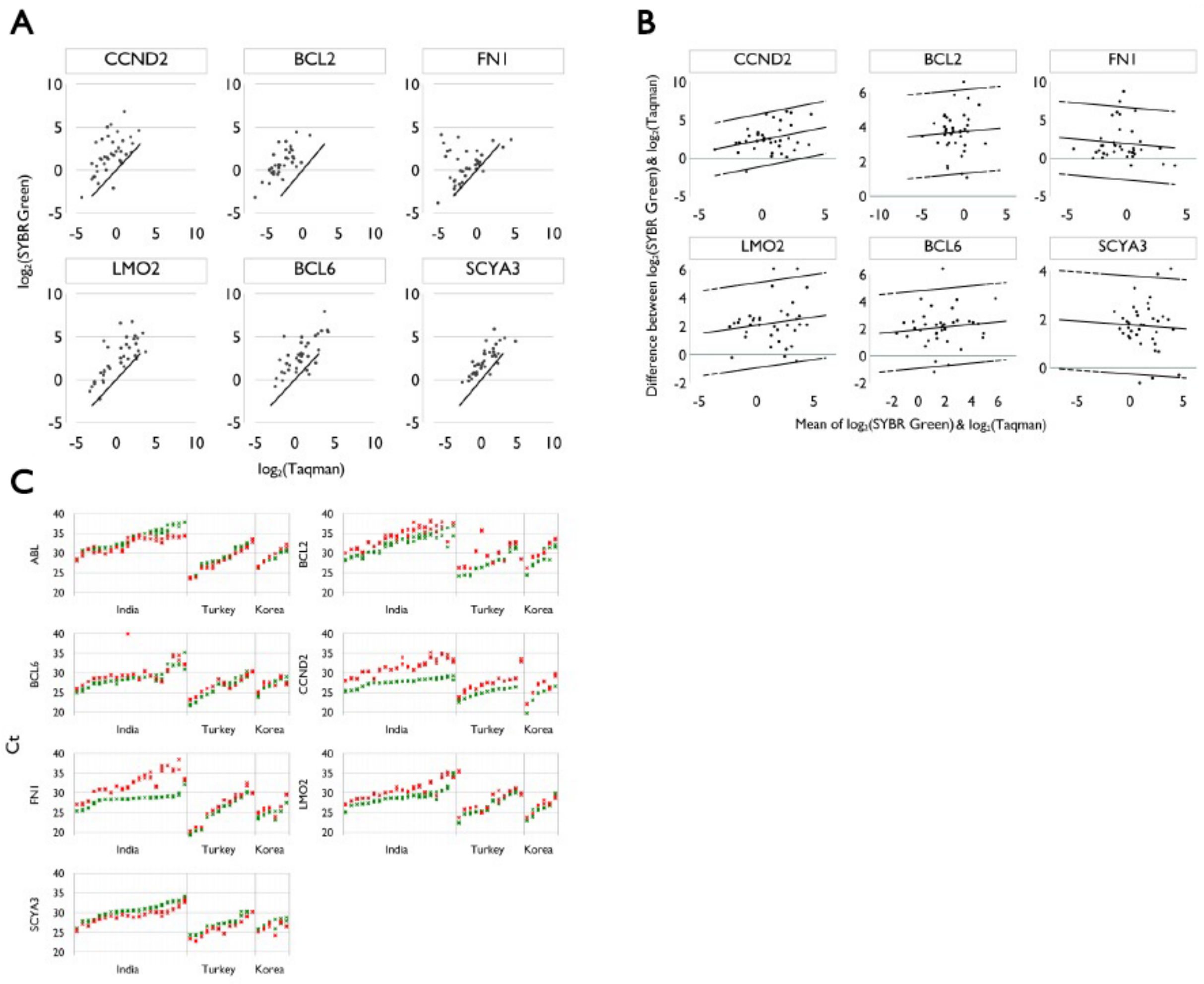

Supplementary Figure S2: Taqman vs SYBR Green Chemistry Comparison. The Data showed high correlation between the both chemistries for the each of the six genes. qRT-PCR results performed with the cDNA samples synthesized with High Volume Synthesis Protocol. **A.** LoA Analysis. **B.** Correlation Analysis. **C.** Comparison with Ct values (SYBR Green is depicted with green crosses, Taqman chemistry depicted with red crosses) using samples from India, Turkey and Korea for the genes shown.

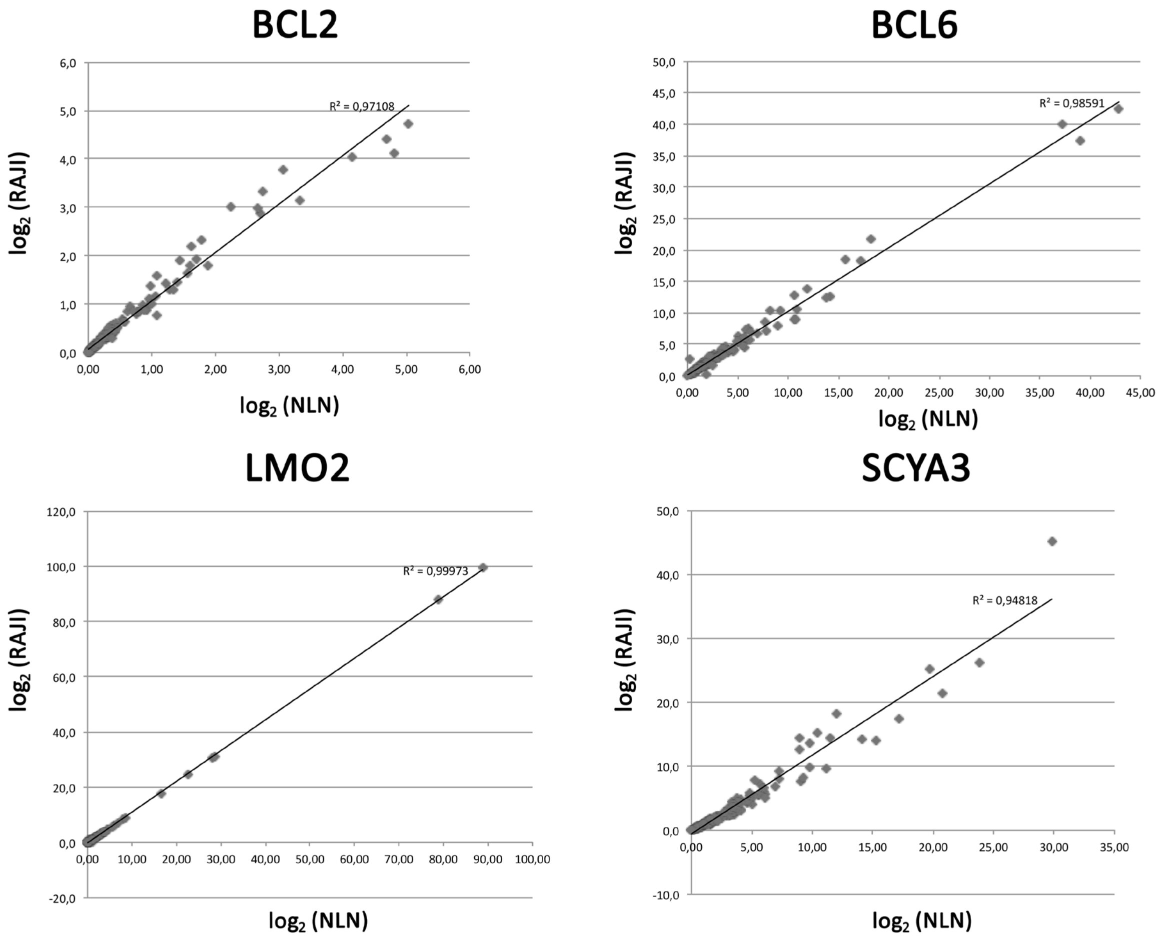

Supplementary Figure S3: Standard Curve Correlation Analysis. NLN vs Raji correlation on BCL2 (r^2=0.97), BCL6 (r^2=0.99), LMO2 (r^2=0.99), SCYA3 (r^2=0.95) expression.

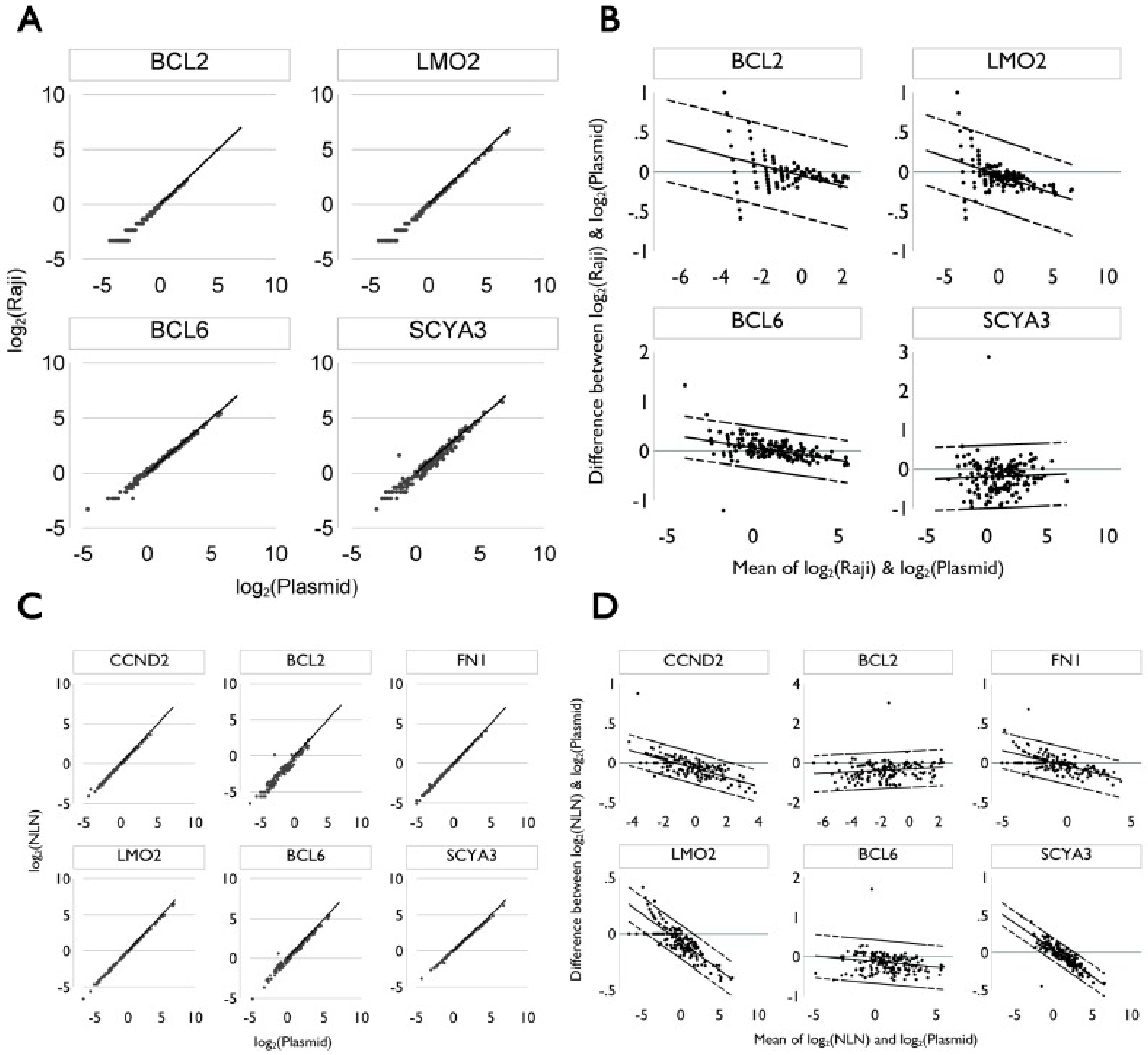

Supplementary Figure S4: Standard Curve Correlation Analysis. The data showed high compatibility between three standard curves from the Raji cell line, plasmids and NLN. **A.** Plasmid vs Raji Correlation on BCL2, LMO2, BCL6 and SCYA3 expression (r^2=0.99 except for SCYA3 (r^2=0.98)). **B.** Plasmid vs Raji LoA on BCL2, LMO2, BCL6 and SCYA3 expression. **C.** Plasmid vs NLN Correlation on six-genes. **D.** Plasmid vs NLN LoA of the six-genes (r^2=0.99).

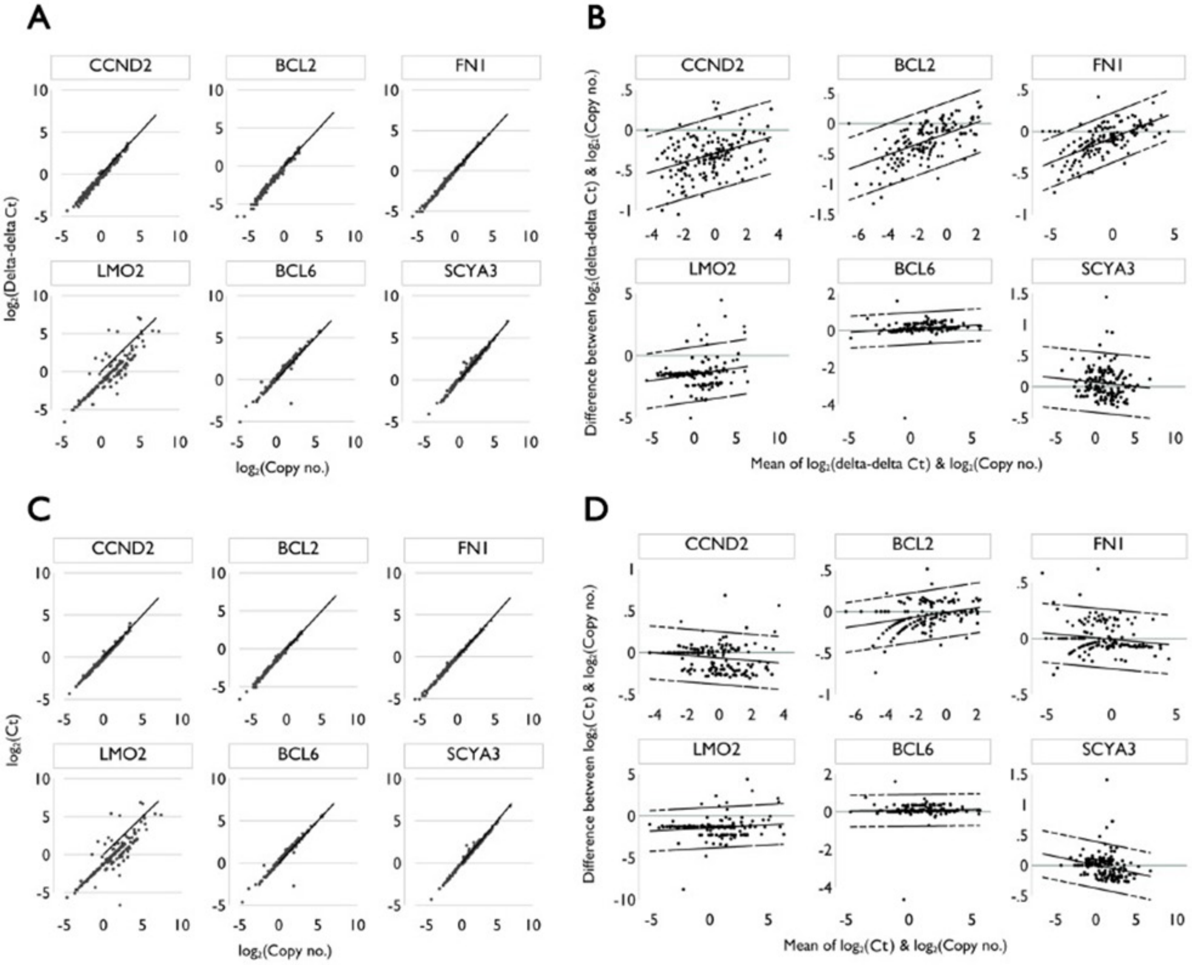

Supplementary Figure S5: Copy Number vs Ct Calculation Comparison. The data shows the concordance between the expression calculation methods. cDNA samples synthesized with High Volume Synthesis Protocol. **A.** Delta Delta Ct vs Copy Number Correlation Analysis (r^2=0.99). **B.** Delta Delta Ct vs Copy Number LoA. **C.** Pfaffl's Equation vs Copy Number Correlation Analysis. **D.** Pfaffl's Equation vs Copy Number LoA: Limits of agreement Analysis (r^2=0.99).

A

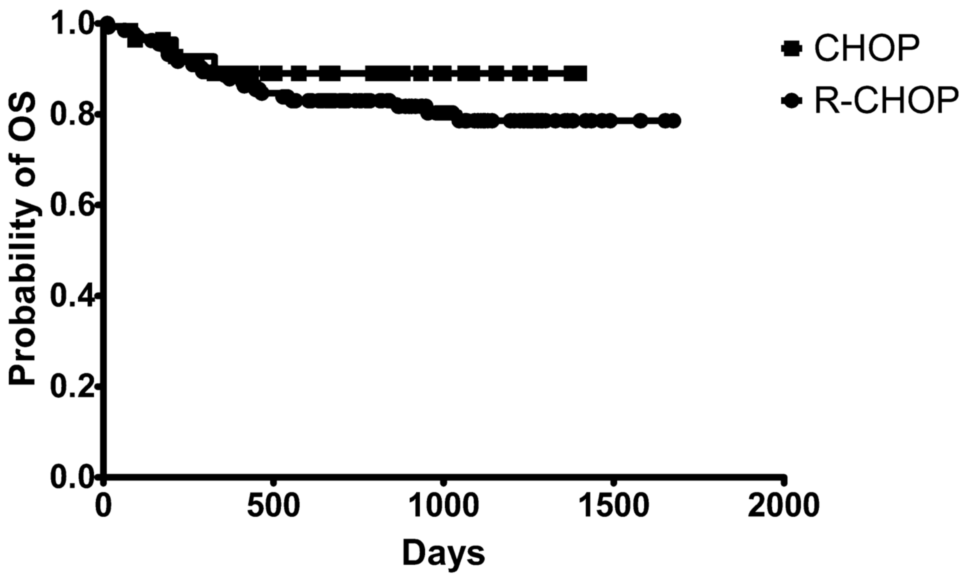

B

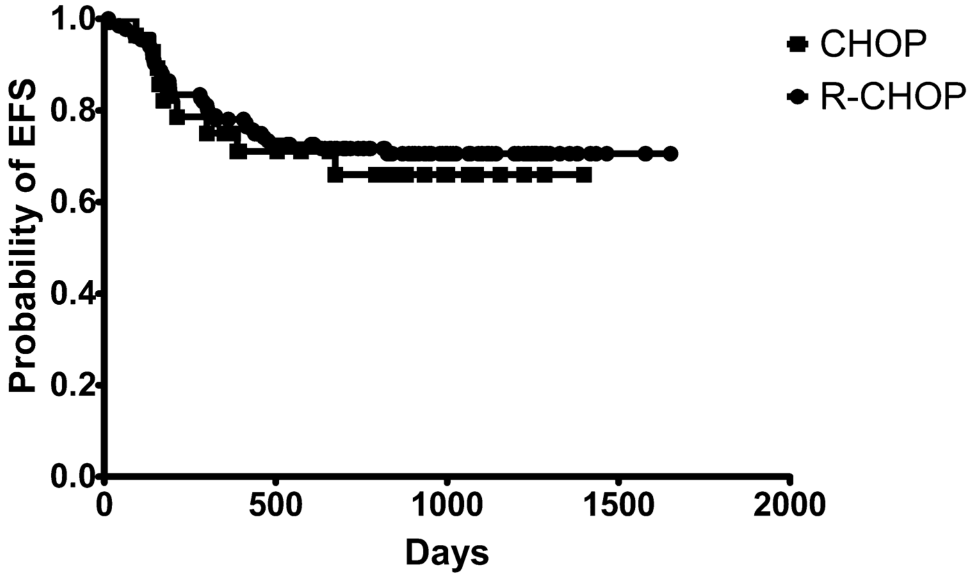

Supplementary Figure S6: Kaplan-Meier survival curve comparison of CHOP versus R-CHOP treatment regimens in terms of overall survival (OS) and event free survival (EFS). No significant difference was observed between CHOP versus R-CHOP treatment in terms of OS (A; p=0.41) or EFS (B; p=0.66), n=28 CHOP, n=134 R-CHOP.

A

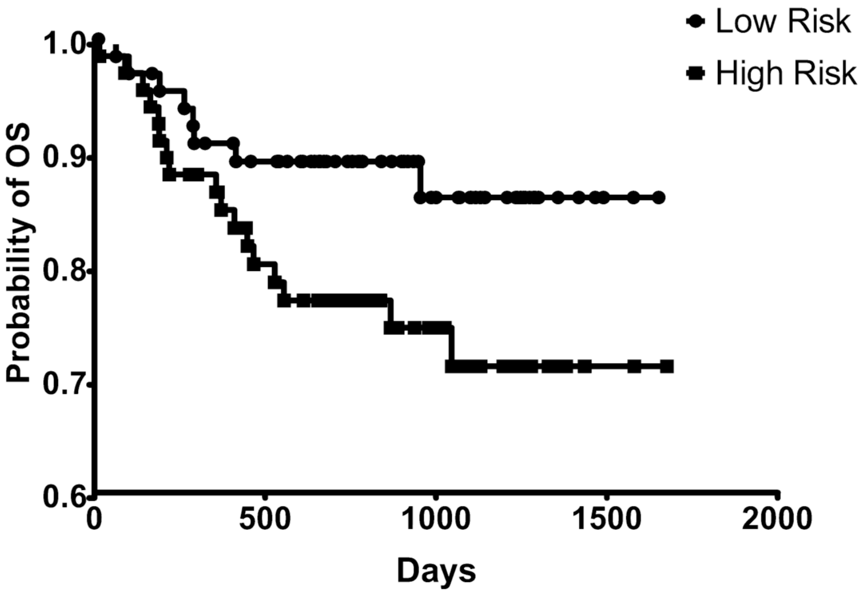

B

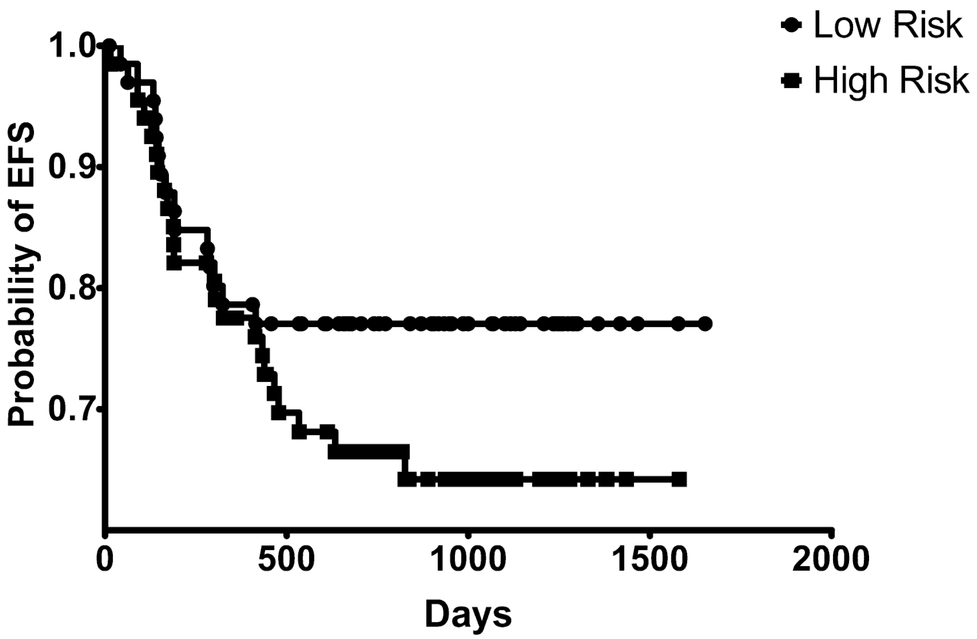

Supplementary Figure S7: Kaplan-Meier survival curve comparison of low vs high risk groups of R-CHOP treated cohort. No Significant difference was observed in the R-CHOP cohort between the low risk (n=67) versus high risk (n=67) 6-gene predictor score in terms of OS (A; p=0.06) and EFS (B; p=0.19).

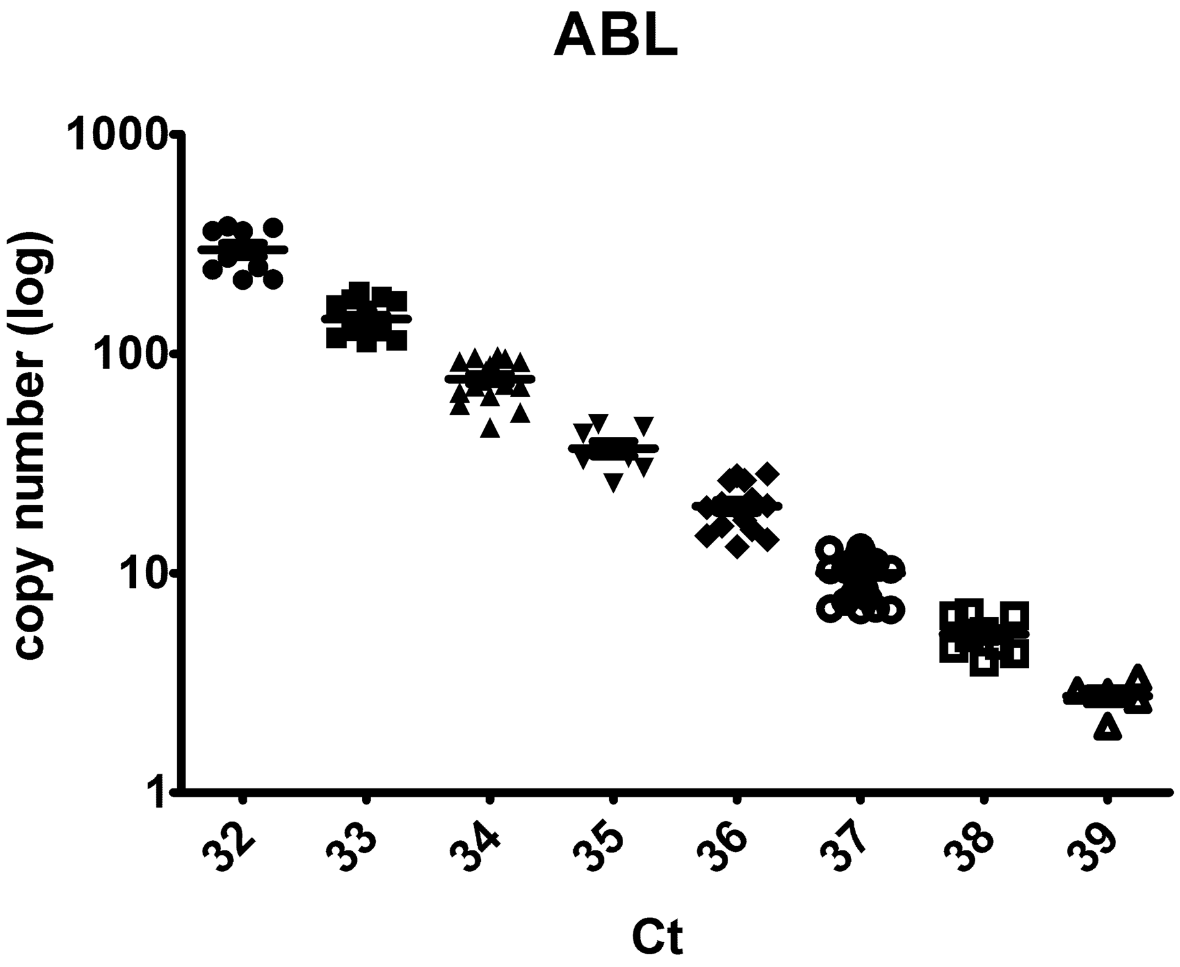

Supplementary Figure S8: Using the plasmid construct (see Materials and Methods) Log Copy number vs Ct of ABL for n=107 patient samples with low volume synthesis showing a linear correlation at the high Ct range.

Supplementary Table S1: Number of samples processed with different cDNA synthesis protocols

cDNA Synthesis protocol	Taqman	SYBR Green
Low Volume Synthesis	63	19
High Volume Synthesis	146	46

Supplementary Table S2: Summary of the qRT-PCR reactions

Countries	Taqman		Sybr Green	
	High volume cDNA	Low volume cDNA	High volume cDNA	Low volume cDNA
Brazil	NA	14	NA	NA
Chile	33	29	NA	NA
Hungary	28	NA	NA	NA
India	22	NA	13	NA
Korea	6	4	6	5
Philippines	13	5	0	0
Thailand	27	9	0	0
Turkey	17	2	11	9
Total Patient Number	**146**	**63**	**30**	**14**

NA; indicates Not Available.

Supplementary Table S3: Cloning Primers for SYBR green reactions

Target Gene	Primer Name	Primer Sequence	Amplicon Size	Annealing Temperature (°C)
ABL	ABLF	ttc agc ggc cag tag cat ctg act t	263	62
	ABLR	gat gta gtt gct tgg gac cca		
BCL2	BCL2F	cgg tgg ggt cat gtg tgt gga	280	60
	BCL2R	ggg gca ggc atg ttg act tca ct		
BCL6	BCL6F	gcc cta caa atg cga aac ctg c	362	64
	BCL6R	tga gaa ggg gct gga gac gaa a		
CCND2	CCND2F	cct tcc gca gtg ctc cta ctt caa	271	62
	CCND2R	ggt gta aat gca cag ctt ctc cgc		
FN1	FN1F	gct tcg gtc agg gtc ggg g	285	62
	FN1R	tgg aaa tgt gag atg gct gtg gtg		
LMO2	LMO2F	cta ctt cct gaa ggc cat cga c	204	60
	LMO2R	att gtc atc tca tag gca cga atc		
SCYA3	SCYA3F	gcc cac att ccg tca cct gct	309	62
	SCYA3R	cct cag gca ctc agc tcc agg t		

Supplementary Table S4: Primer sequences and PCR conditions of used SYBR green qRT-PCR

Target Gene	Primer Name	Primer Sequence	Amplicon Size	Annealing Temperature (°C)
ABL	ENF1003	tgg aga taa cac tct aag cat aac taa agg t	124	60
	ENR1063	gat gta gtt gct tgg gac cca		
BCL2	BCL2-F	acc tgc aca cct gga tcc a	96	60
	BCL2-R	aca gcc agg aga aat caa aca ga		
BCL6	BCL6-F	gcg aat cca cac agg aga gaa	63	60
	BCL6-R	ttg tga cgg aaa tgc agg tta c		
CCND2	CCND2-F	ccc tac atg cgc aga atg gt	72	60
	CCND2-R	gac ctc ttc ttc gca ctt ctg ttc		
FN1	FN1-F	cta tgg ccg tgg cat tgg	112	60
	FN1-R	gtg gga gtt ggg ctg act		
LMO2	LMO2-F	caa act ggg ccg gaa gct	68	60
	LMO2-R	atg cgc aga gac cgt ctt g		
SCYA3	SCYA3-F	atg gct ctc tgc aac cag ttc t	53	60
